# A new technique for low back pain in lumbar disc herniation: percutaneous endoscopic lumbar discectomy combined with sinuvertebral nerve ablation

**DOI:** 10.1186/s13018-024-04831-8

**Published:** 2024-06-08

**Authors:** Yanjun Huang, Shangshu Wei, Yanzhu Shen, Sizheng Zhan, Ping Yi, Xiangsheng Tang

**Affiliations:** 1 Chinese Academy of Medical Sciences & Peking Union Medical College, Beijing, China; 2 Department of Orthopaedics, China-Japan Friendship Hospital, Beijing, China

**Keywords:** Low back pain, Sinuvertebral nerve, Lumbar disc herniation, Percutaneous endoscopy, Case series

## Abstract

**Background:**

Percutaneous endoscopic lumbar discectomy (PELD) has demonstrated efficacy in alleviating leg pain among patients with lumbar disc herniation. Nonetheless, residual back pain persists as a troubling issue for surgeons following the procedure. In the treatment of discogenic back pain, sinuvertebral nerve radiofrequency ablation has shown promising results. Nevertheless, the potential benefit of simultaneously implementing sinuvertebral nerve radiofrequency ablation during PELD surgery to address residual back pain has not been thoroughly investigated in current literature.

**Methods:**

This retrospective study reviewed Lumbar disc herniation (LDH) patients with low back pain who underwent combined PELD and sinuvertebral nerve ablation in our department between January 2021 and September 2023. Residual low back pain post-surgery was assessed and compared with existing literature.

**Results:**

A total of 80 patients, including 53 males and 27 females, were included in the study. Following surgical intervention, patients demonstrated remarkable improvements in pain and functional parameters. One month post-operatively, the VAS score for low back pain exhibited a 75% reduction (6.45 ± 1.3 to 1.61 ± 1.67), while the VAS score for leg pain decreased by 85% (7.89 ± 1.15 to 1.18 ± 1.26). Notably, the JOA score increased from 12.89 ± 5.48 to 25.35 ± 4.96, and the ODI score decreased form 59.48 ± 9.58 to 20.3 ± 5.37. These improvements were sustained at three months post-operatively. According to the modified Mac Nab criteria, the excellent and good rate was 88.75%. Residual low back pain is observed to be comparatively reduced compared to the findings documented in earlier literature.

**Conclusion:**

The combination of percutaneous endoscopic lumbar discectomy and sinuvertebral nerve ablation demonstrates effective improvement in low back pain for LDH patients.

## Introduction

Lumbar disc herniation (LDH) is a prevalent degenerative condition affecting the lumbar spine with an annual incidence ranging from 0.1 to 0.5% and a lifetime incidence of approximately 1–2% [1]. LDH presents with symptoms such as sciatica, lower back and leg pain, and limited mobility, significantly reducing patients’ quality of life and imposing a substantial socioeconomic burden [2]. The pathogenesis of lumbar disc herniation involves physical compression, release of inflammatory factors [3], and nerve irritation, ultimately affecting nerve conduction. Among the nerves associated with the intervertebral disc, the sinuvertebral nerve is considered the primary mediator of low back pain [4].

Conventional treatment modalities for lumbar disc herniation, including bed rest, physical therapy, and medication [5], often yield unsatisfactory outcomes. In recent years, there has been rapid development in endoscopic techniques for spinal interventions, which have emerged as highly effective approaches for managing intervertebral disc diseases [6]. In 1991, Kambin introduced the concept of “Kambin’s triangle” and reported the surgical technique of endoscopic lumbar disc removal [7], which has since become a cornerstone of spine endoscopic surgery. Percutaneous endoscopic lumbar discectomy (PLED) aims to alleviate symptoms resulting from nerve compression, such as cauda equina syndrome and sciatica, by removing the protruding disc tissue [8–10]. In follow-up studies by Xu et al., out of 113 patients who underwent PELD surgery, none reported residual leg pain within two years, although 32 cases displayed residual low back pain [11]. Similarly, Zhong et al. reported that out of 355 patients who underwent PELD, 130 experienced varying degrees of low back pain after the procedure [12]. Another retrospective review involving 88 PELD-treated patients also indicated that 21.6% experienced low back pain post-surgery [13]. While PELD demonstrates high effectiveness in alleviating leg pain and sciatica, the management of low back pain remains suboptimal.

We posit that the adjunctive use of sinuvertebral nerve ablation with percutaneous endoscopic lumbar discectomy (PELD) yields superior outcomes, particularly in alleviating low back pain, compared to PELD alone. To investigate this hypothesis, we conducted a comparison of existing literature to assess the therapeutic efficacy of combining PELD with sinuvertebral nerve ablation in the treatment of low back pain associated with lumbar disc herniation.

## Methods

### Research design and study population

This retrospective study aimed to investigate patients with low back pain who underwent percutaneous endoscopic surgery at the Department of Orthopaedics in the China-Japan Friendship Hospital between January 2021 and September 2023. The study received approval from the Clinical Research Ethics Committee of the China-Japan Friendship Hospital (2022-KY-104), and informed consent was obtained from all participating patients.

The inclusion criteria were as follows: (1) adult patients, (2) presence of typical symptoms indicating nerve root compression, (3) confirmation of segmental disc herniation at the corresponding level through imaging examinations, (4) presence of typical dull low back pain or lumbosacral pain symptoms, and (5) failure of conservative treatment (e.g., rest, medication, physical therapy, muscle exercises) for a duration of 3 months. The exclusion criteria included: (1) patients with other spinal diseases, (2) patients with low back pain caused by other etiologies, (3) patients with contraindications for surgery, (4) patients who declined surgery, (5) patients lost to follow-up, (6) patients underwent percutaneous endoscopic interlaminar discectomy(PEID).

### Assessment criteria

The assessment of treatment outcomes included the evaluation of visual analog scale (VAS) scores for low back pain and leg pain, Japanese Orthopaedic Association (JOA) scores, and Oswestry Disability Index (ODI) scores before surgery, at the 1-month and 3-months postoperative follow-up. The modified Mac Nab criteria were used to assess the final treatment outcomes.

### Surgical procedure

All surgical procedures were conducted by a experienced physician at the China-Japan Friendship Hospital who possessed expertise in percutaneous endoscopic techniques. Use antibiotics 30 min before surgery to prevent infection.

The patients were positioned in the prone position. Local anesthesia was administered to all patients, involving the skin and subcutaneous tissue, muscles and fascia, facet joints, and intervertebral disc surfaces. The 18G puncture needle was inserted approximately 6–10 cm outside the spinous process on the surgical side, targeting the base of superior articular process. Intraoperative fluoroscopy was employed to verify precise needle placement. A skin incision of approximately 0.8 cm in diameter was made around the guide needle, followed by sequential insertion of dilators to establish a soft tissue corridor. Subsequently, the working channel was placed within the soft tissue corridor, and fluoroscopy was used to confirm its positioning at the target site before inserting the endoscope. The ventral bone of the superior articular process was removed for the foraminoplasty. If necessary, the foraminoplasty may be performed several times under visual inspection to deal with the herniated disc and the lateral recess.

**Fig. 1:**
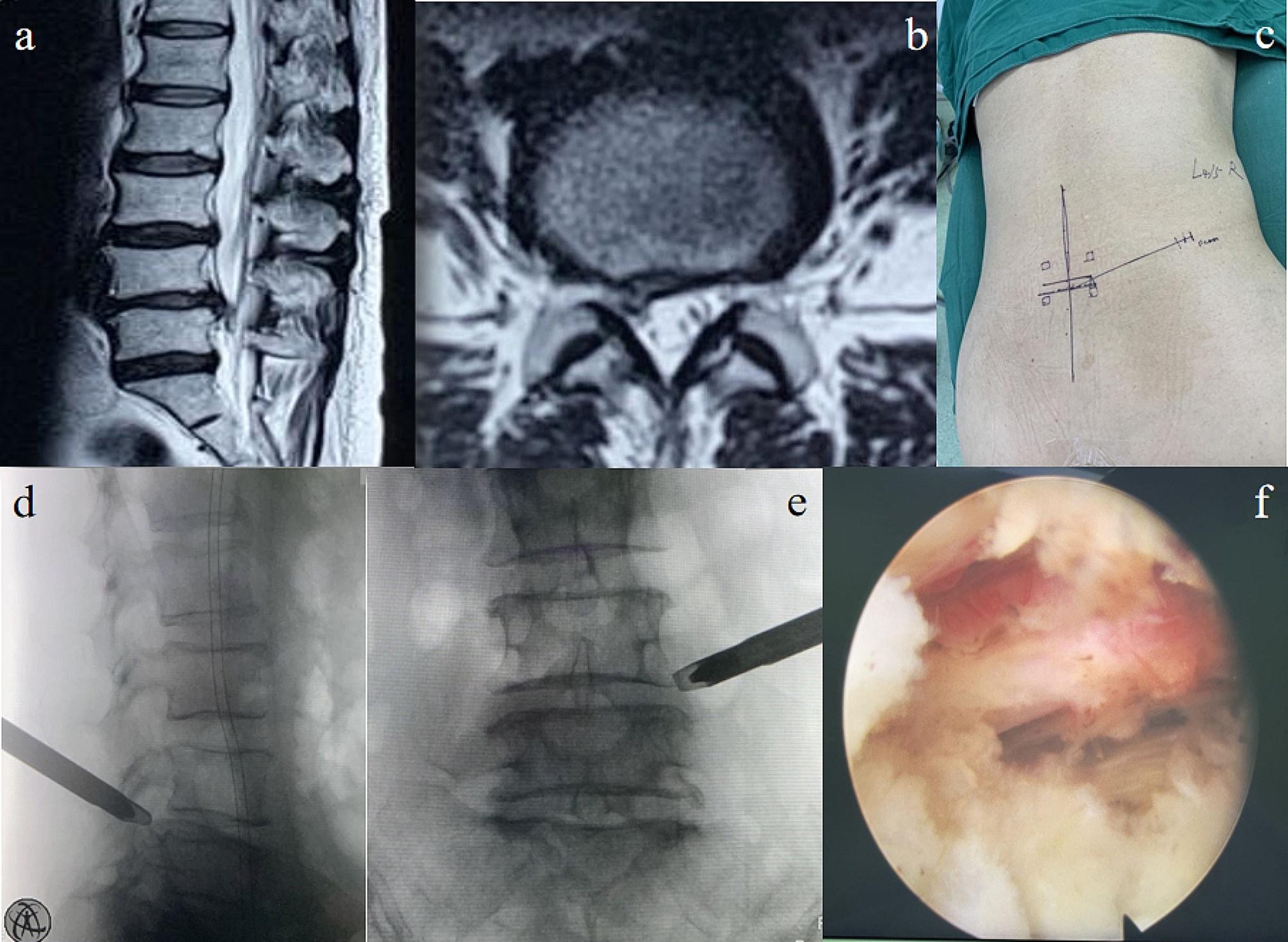
Images during treatment: (**a, b**) herniated intervertebral disc of L4/5 segment can be seen in the preoperative T2-weighted MRI; (**c**) surgical entry site markings; (**d, e**) Intraoperative fluoroscopy of the working channel of spinal endoscopy; (**f**) the view under the spinal endoscopy showed the ventral dura during the surgery

During the exposure of the intervertebral disc, flexible bipolar radiofrequency was employed to clean the distribution area of the sinuvertebral nerve from the outside to the inside, while the exiting nerve root was intentionally kept outside the protective cannula. The nucleus pulposus tissue was removed with a nucleus forceps, and the annulus fibrosus was probed. Confirm from the inside to outside that the distribution of the sinuvertebral nerve has been dissected and the herniated nucleus pulposus was completed removal. The partial annulotomy was performed if necessary. Finally, the endoscope was withdrawn, and the working channel was removed, followed by layered closure of the incision. For a visual reference, please refer to Fig. 1.

Postoperatively, patients received symptomatic treatments to reduce edema, alleviate pain, prevent infection, and promote nerve nutrition. Starting from the day following surgery, patients were encouraged to mobilize while wearing a lumbar support brace, which was worn continuously for 4 weeks. Patients were also instructed to gradually engage in exercising their back muscles.

### Statistical analysis

Data analysis was conducted using R (version 4.2.1) and RStudio (version 2022.07.1 Build 554). Normally distributed continuous variables were presented as mean ± standard deviation (SD), and categorical variables were presented as percentages. The permutation-t-test was employed to assess the differences in the data. A two-tailed *p*-value < 0.01 was considered statistically significant.

## Results

This study enrolled a cohort of 80 patients, with an average age of 47.8 ± 16.8 years, comprising 53 males and 27 females. Baseline characteristics of patients can be found in the Table 1. All patients underwent the planned surgical intervention. The Visual Analog Scale (VAS) score for low back pain presented as 6.45 ± 1.3 preoperatively, decreasing to 1.61 ± 1.67 at 1 month post-surgery, and further declining to 1.37 ± 1.44 at 3 months post-surgery. Similarly, VAS scores for leg pain showed a reduction from 7.89 ± 1.15 preoperatively to 1.18 ± 1.26 at 1 month post-surgery, and 1.01 ± 1.07 at 3 months post-surgery. The Japanese Orthopaedic Association (JOA) scores improved from 12.89 ± 5.48 preoperatively to 25.35 ± 4.96 at 1 month, and 27.23 ± 5.02 at 3 months post-surgery. Furthermore, the Oswestry Disability Index (ODI) scores decreased from 59.48 ± 9.58 preoperatively to 20.3 ± 5.37 at 1 month postoperatively, and 18.92 ± 5.11 at 3 months post-surgery. The postoperative scores displayed marked improvement compared to preoperative values (*P* < 0.01). Postoperative evaluations at 1 month and 3 months revealed significant reductions in pain severity. Specifically, the Visual Analog Scale (VAS) scores for low back pain exhibited a 75% and 79% decline, respectively, compared to preoperative levels. Similarly, the VAS scores for leg pain demonstrated a marked decrease of 85% and 87% at 1 month and 3 months, respectively, relative to the preoperative period.

**Fig. 2:**
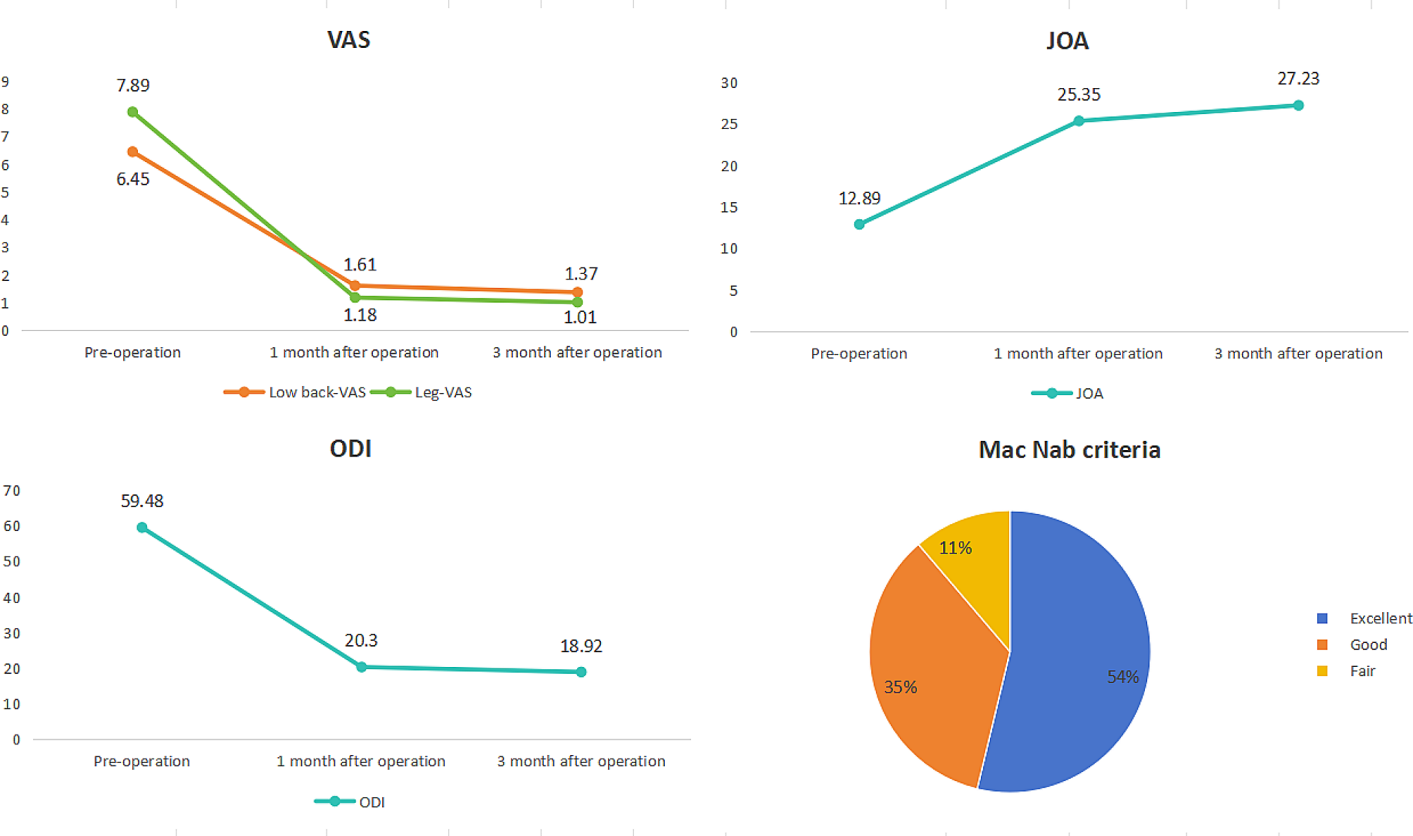
improvement in postoperative VAS, JOA, and ODI scores and proportion of outcomes according to the modified Mac Nab criteria

Evaluation of the treatment outcomes at one month postoperatively, according to the modified Mac Nab criteria, revealed excellent results in 43 cases, good outcomes in 28 cases, and fair outcomes in 9 cases. The combined excellent and good rate was 88.75%. Detailed evaluation results can be found in the Table 2; Fig. 2 below.

**Table 1:** Baseline characteristics of patients with LDH

	Patients (*n* = 80)
Age, years	47.8 ± 16.8 (range 19–85)
Sex	
Male	53(66.25)
Female	27(33.75)
LDH segment distribution	
L3/L4	7 (8.75)
L4/L5	62 (77.5)
L5/S1	11 (13.75)
Lesion site	
Central	17 (21.25)
Left paracentral	31 (38.75)
Right paracentral	32 (40)
Physical examination	
Lasegue sign positive	63 (78.75)
Lower limb hypesthesia	35 (43.75)

**Table 2:** Outcomes of accessment

	Patients (*n* = 80)
Low back-VAS	
Pre-operation	6.45 ± 1.3
1 month after operation	1.61 ± 1.67
3 month after operation	1.37 ± 1.44
Leg-VAS	
Pre-operation	7.89 ± 1.15
1 month after operation	1.18 ± 1.26
3 month after operation	1.01 ± 1.07
JOA	
Pre-operation	12.89 ± 5.48
1 month after operation	25.35 ± 4.96
3 month after operation	27.23 ± 5.02
ODI	
Pre-operation	59.48 ± 9.58
1 month after operation	20.3 ± 5.37
3 month after operation	18.92 ± 5.11
Mac Nab criteria	
Excellent	43 (53.25)
Good	28 (35)
Fair	9 (11.15)

## Discussion

Back pain is a prevalent medical condition worldwide, affecting approximately 80% of individuals at least once in their lifetime [14]. Among the various causes of back pain, degenerative changes in the intervertebral discs are considered a primary factor, accounting for 26-42% of cases [15]. Extensive research has been conducted on back pain resulting from disc herniation. The onset of back pain is associated with disc aging and decreased water content, followed by a chronic inflammatory response [16, 17]. Inflammatory mediators contribute to the deterioration of the intervertebral disc structure, leading to annular fibrosis [18]. Additionally, they promote the formation of new blood vessels, nerve innervation, and sensitization, ultimately resulting in pain transmission [19]. Based on anatomical studies, Chen et al. have proposed a significant role for the sinuvertebral nerve in back pain transmission [20].

Percutaneous endoscopic discectomy has demonstrated partial alleviation of clinical symptoms associated with lumbar disc herniation, particularly in cases of sciatica and leg pain. However, its efficacy in managing low back pain remains suboptimal. A study involving 249 patients [21] reported a decline in the mean Visual Analog Scale (VAS) score for low back pain from 4.44 to 1.64 at four weeks postoperatively, representing a reduction of approximately 63.1%. Similarly, in a study of 56 patients [22], the mean VAS score decreased from 3.49 to 1.39 at 12 months postoperatively, indicating a reduction of approximately 60.1%. Moreover, in a study of 167 patients [23], the mean VAS score for low back pain decreased from 4.38 to 1.49 at two years postoperatively, reflecting a decrease of about 66%. In comparison with previous research, our approach offers advantages in addressing low back pain in patients with lumbar disc herniation, as evidenced by the 75% reduction from 6.45 to 1.61 in the mean VAS score for low back pain observed in the 80 patients one month after surgery.

Significant success has also been achieved through the targeting of sinuvertebral nerves. For instance, Kim et al. performed sinuvertebral nerve radiofrequency ablation on 30 patients, resulting in an average 73% decrease in back pain visual analog scale (VAS) scores six months post-surgery, with a surgical excellent and good rate of 93% [24]. In another study, Liu et al. conducted a retrospective analysis of 32 patients who underwent sinuvertebral nerve block. They observed a decrease in the average VAS score for back pain from 5.75 to 2.5 three days after the procedure, along with a reduction in the Oswestry Disability Index (ODI) score from 32.59 to 17.28 [25]. Furthermore, Koreckij et al. performed basivertebral nerve radiofrequency ablation on 58 patients, achieving pain relief of over 50% in 72.4% of patients and a pain-free rate of 31.0% two years post-surgery [26].

The sinuvertebral nerve originates from the ventral spinal nerves and re-enters the spinal canal through the intervertebral foramen. It supplies the posterior aspect of the intervertebral disc, the posterior longitudinal ligament, vertebral bodies, pedicles, and the relevant soft tissues of the anterior part of the intervertebral foramen and spinal canal [27]. Comprising both somatic nerve branches from the spinal nerves and sympathetic nerve branches from the gray communicating rami, the sinuvertebral nerve is divided into main and collateral branches [28]. These branches originate from the lateral aspect of the intervertebral disc, with the main branch passing below the pedicle and dividing into ascending, transverse, descending, and spinal canal branches within the intervertebral foramen. While the main branch supplies almost all areas within the spinal canal except the medial side of the intervertebral disc, the collateral branch mainly innervates the lateral aspect of the intervertebral disc and the lower edge of the pedicle [20].

**Fig. 3:**
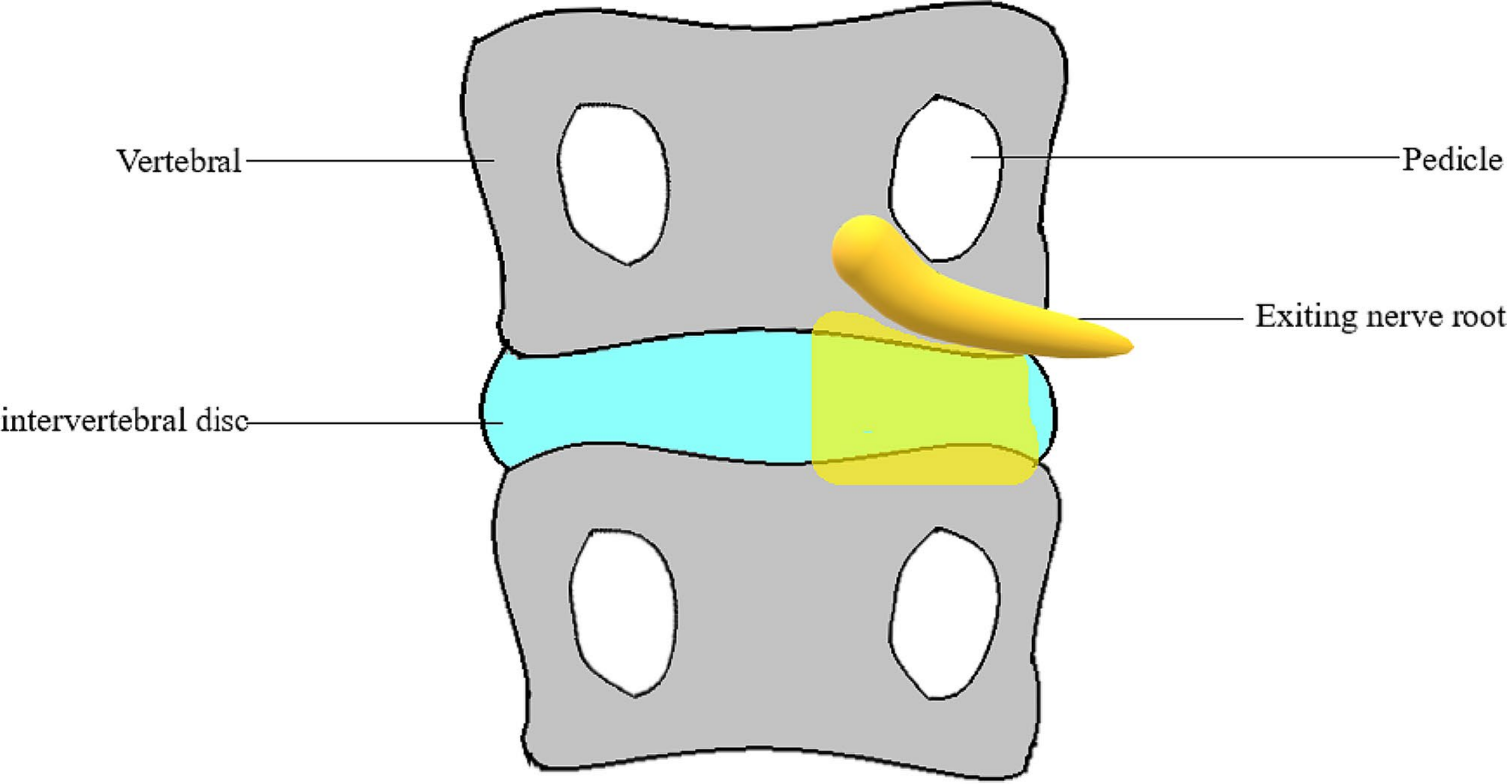
Visual representation, the highlight is the scope of ablation

Identifying the sinuvertebral nerve is challenging even under endoscopy due to its delicate nature. Therefore, drawing on previous research, we employed an anatomical localization method to determine the extent of ablation. This approach involved clearing the surface area of the intervertebral disc, the area above the intervertebral disc, and the inner margin of the nerve root, effectively blocking the main and collateral branches of most sinuvertebral nerves. By avoiding the initial region of the sinuvertebral nerve, we minimized the risk of damaging critical tissues such as ganglia and arteries, thereby significantly reducing complications. The ablation range spanned the intervertebral disc as its central axis. The outermost perimeter of the ablation encompassed the outer edge of the foramen. The innermost limit of the ablation encompassed the midline of the vertebral body. The superior boundary of the ablation coincided with the lower endplate of the upper vertebral body. The inferior boundary of the ablation coincided with the upper endplate of the lower vertebral body. Figure 3 provides a visual representation of the scope of ablation.

Variations in surgical proficiency and conceptual understanding among surgeons can lead to disparate outcomes. However, in our study, all surgical procedures were performed by the same experienced specialist, ensuring a high degree of technical consistency. What’s more, to further standardize and homogenize the surgical interventions, we employed a uniform surgical procedure and similar ablation scope for each included patient.

The study’s advantages notwithstanding, several limitations deserve attention. Notably, for patients whose lumbar disc herniations of a specific size, shape, and location, our actual ablation scope may have been more extensive than described above, potentially enhancing the effective rate and introducing a positive bias in efficacy estimates. Secondly, we only blocked the sinuvertebral nerve on one side, while pain can still be transmitted to the contralateral side through anastomotic branches. Blocking both sides may yield better outcomes. Additionally, the surgical excision of fascia, muscles, ligaments, and bone tissue may contribute to postoperative low back pain [29]. Moreover, as a retrospective study, we lacked a control group, which prevented us from establishing its comparative effectiveness with other treatment methods. Lastly, the sample size was limited, and the follow-up period was relatively short.

This study represents a pivotal step in the advancement of the understanding of sinuvertebral nerve ablation. In the future, large-sample, multicenter randomized controlled studies hold the promise of enhancing the reliability of our findings. Currently, we rely primarily on literature of anatomical researches to guide the ablation range for sinuvertebral nerves, and the optimal ablation range remains to be verified through clinical studies. The limitations of the current intervertebral foraminoscopy technique have restricted us to ablating only a portion of the branches of the sinuvertebral nerve. We eagerly anticipate the emergence of novel surgical techniques that will enable us to ablate additional branches responsible for pain transmission while minimizing tissue damage.

## Conclusion

Despite the nascent nature of this preliminary exploration into novel technologies, the limitations inherent to the study are undeniable. Notwithstanding these limitations, the observational evidence gleaned from this study unequivocally demonstrates the efficacy and substantial potential of percutaneous endoscopic lumbar discectomy coupled with sinuvertebral nerve ablation in alleviating low back pain. Given the aforementioned findings, patients suffering from lumbar disc herniation and presenting with low back pain as the predominant symptom, or moderate to severe low back pain, may elect this surgical treatment as an alternative therapy to alleviate their pain and improve their quality of life.

## Data Availability

The study received approval from the Clinical Research Ethics Committee of the China-Japan Friendship Hospital (2022-KY-104), and informed consent was obtained from all participating patients.
